# Strong correlation of lumefantrine concentrations in capillary and venous plasma from malaria patients

**DOI:** 10.1371/journal.pone.0202082

**Published:** 2018-08-16

**Authors:** Liusheng Huang, Norah Mwebaza, Richard Kajubi, Florence Marzan, Camilla Forsman, Sunil Parikh, Francesca T. Aweeka

**Affiliations:** 1 Drug Research Unit, Department of Clinical Pharmacy, University of California, San Francisco, CA, United States of America; 2 Department of Pharmacology and Therapeutics, Makerere University College of Health Sciences, Kampala, Uganda; 3 Infectious Diseases Research Collaboration, Kampala, Uganda; 4 Department of Epidemiology of Microbial Diseases, Yale School of Public Health, New Haven, CT, United States of America; Universidade de Sao Paulo Instituto de Ciencias Biomedicas, BRAZIL

## Abstract

**Background:**

Lumefantrine is a long-acting antimalarial drug with an elimination half-life of over 3 days and protein binding of 99 percent. Correlation of lumefantrine concentrations from capillary plasma via fingerprick (C_c_) versus venous plasma (C_v_) remains to be defined.

**Methods:**

Venous and capillary plasma samples were collected simultaneously from children, pregnant women, and non-pregnant adults at 2, 24, 120hr post last dose of a standard 3-day artemether-lumefantrine regimen they received for uncomplicated malaria. Some of the enrolled children and pregnant women were also HIV-infected. Samples were analyzed via liquid chromatography tandem mass spectrometry. Linear regression analysis was performed using the program Stata® SE12.1.

**Results:**

In children, the linear regression equations for C_c_ vs C_v_ at 2, 24, and 120hr (day 7) post dose are [C_c_] = 1.05*[C_v_]+95.0 (n = 142, R^2^ = 0.977), [C_c_] = 0.995*[C_v_]+56.7 (n = 147, R^2^ = 0.990) and [C_c_] = 0.958*[C_v_]+18.6 (n = 139, R^2^ = 0.994), respectively. For pregnant women, the equations are [C_c_] = 1.04*[C_v_]+68.1 (n = 43, R^2^ = 0.990), [C_c_] = 0.997*[C_v_]+37.3 (n = 43, R^2^ = 0.993) and [C_c_] = 0.941*[C_v_]+11.1 (n = 41, R^2^ = 0.941), respectively. For non-pregnant adults, the equations are [C_c_] = 1.05*[C_v_]-117 (n = 32, R^2^ = 0.958), [C_c_] = 0.962*[C_v_]+9.21 (n = 32, R^2^ = 0.964) and [C_c_] = 1.04*[C_v_]-40.1 (n = 32, R^2^ = 0.988), respectively. In summary, a linear relationship with a slope of ~1 was found for capillary and venous lumefantrine levels in children, pregnant women and non-pregnant adults at 2hr, 24hr and 120hr post last dose, representing absorption, distribution, and elimination phases.

**Conclusions:**

Capillary and venous plasma concentration of lumefantrine can be used interchangeably at 1:1 ratio. Capillary sampling method via finger prick is a suitable alternative for sample collection in clinical studies.

## Introduction

Lumefantrine (LF) previously named benflumetol is an antimalarial drug synthesized in 1970s in China [1]. LF is currently used as the long-acting partner drug of artemether, and co-formulated artemether-lumefantrine (AL) is the most widely used first-line artemisinin-based combination therapy in sub-Saharan Africa, where malaria transmission, morbidity, and mortality rates are the highest in the world [2]. A significant number of AL recipients are children, and particularly in this group, venous blood sampling is not convenient in rural settings. An alternative approach is capillary blood sample collection via finger or heel stick that greatly facilitates sample collection. In addition, application of mass spectrometry to drug quantification in the past two decades has enabled the development of methods with high sensitivity, making small volume sampling approaches feasible. Field studies have expanded use of these sampling methods [3, 4].

Drug levels in capillary plasma may differ from levels in venous plasma [5], depending on pharmacokinetic properties of the drug. Although the majority of historical LF pharmacokinetic data was based on quantification from venous plasma, current research using an intensive pharmacokinetic design often depends on a merging of venous and capillary measurements. Moreover, comparisons are made of population pharmacokinetic results dependent on capillary measurements to results in the literature based on venous measurements. Thus, to properly interpret and analyze newer data emerging from capillary sampling or based on a composite of capillary and venous concentrations, correlation between capillary and venous LF level needs to be established. To our knowledge, very few correlation studies have been reported for LF [6, 7]. The 1^st^ study excluded pregnant or lactating women and children under 2 years old. The 2^nd^ study was performed in pregnant and non-pregnant women but capillary samples were only collected from pregnant women, the concentrations of venous LF and capillary LF were not paired. Herein, we reported an extensive correlation study of capillary versus venous plasma levels of LF at 3 time intervals (2, 24, and 120hr post last dose) in 3 different demographic groups: children, pregnant women and non-pregnant adults, considering pharmacokinetic phases and physiological distinctions that may impact pharmacokinetic parameters in these demographic groups. Our study was part of a larger study evaluating the impact of antiretrovirals for HIV, age and pregnancy on AL pharmacokinetics and pharmacodynamics. The study was registered at clinicaltrials.gov as NCT01717885 [8] and reported previously [9–11].

## Methods

### Clinical study overview

The parent study was conducted in Tororo, Uganda from 5 August 2011 to December 29, 2014 with sample analysis completed in December, 2015 to investigate the pharmacokinetics and pharmacodynamics of AL in HIV-infected and HIV-uninfected children and pregnant women with uncomplicated *P*. *falciparum* malaria. Participants were children (eligible age, 0.5-8yr), pregnant women, and non-pregnant adults with uncomplicated *P*. *falciparum* malaria. Children and pregnant women co-infected with HIV were stabilized with either efavirenz, lopinavir/ritonavir, or nevirapine-based antiretroviral therapy. Grapefruit juice and any drugs known to affect CYP450 metabolism were not allowed, except for the antiretrovirals specified in this study. Adult (pregnant and non-pregnant) participants underwent a standard 6-dose regimen (4 tablets of Coartem 20 mg/120 mg, Novartis Pharma AG, Basel, Switzerland) over 3 days administrated with 200mL milk to enhance and control for lumefantrine absorption. Pediatric participants underwent weight-based 6-dose regimen over 3 days. Dispersible AL tablets were administered with milk or breastfeeding based on the following guidelines: weight <15kg, 1 tablet; 15-25kg, 2 tablets; 25-35kg, 3 tablets; ≥35kg, 4 tablets.

### Clinical samples collection

Intensive pharmacokinetic sampling started on day 3 following the last dose (the 6^th^ dose), with the “day 7” sample occurring 120 hours after the last dose. It was actually day 8 in this study due to the dosing strategy adapted for PK sampling. Venous and capillary samples were collected simultaneously at 2, 24, and 120hr post last dose of standard six-dose regimen of AL from children, pregnant women and non-pregnant adults with malaria enrolled in the clinical trial based in a high-transmission region of Uganda [8]. Capillary samples (~200 μL) were collected via finger prick and venous samples (500 μL) were collected via an indwelling intravenous catheter. All blood samples were collected in K_3_EDTA-coated tubes, immediately placed on ice and centrifuged at 800 g for 10 min at 4°C. Plasma was separated and stored at -80°C until analysis. The study was approved by the Uganda National Council for Science and Technology, the Makerere University School of Medicine Research and Ethics Committee, the Yale Human Investigations Committee, and the University of California, San Francisco Committee on Human Research. All participants or their guardians read and signed the informed consent before participation.

### Sample analysis

The collected plasma samples were analyzed with a liquid chromatography tandem mass spectrometry system. The method was validated and published previously [12]. The method was sensitive with the lower limit of quantification (LLOQ) at 50 ng/mL and only 25 μL samples were required in the method. The method was also cross-validated with another in-house method based on high performance liquid chromatography coupled with ultra-violet/visible spectrophotometry [13].

### Statistical data analysis

Data analysis was performed using STATA® SE12.1. The relationship between capillary and venous plasma LF levels was modelled using a linear relationship with estimated intercept and slope. The linear least squares regression models were built using concentrations or logarithm-transformed concentrations and the final models were selected based on maximal coefficient of determination (R^2^) and visual check.

## Results

### Sample profile

These samples were collected during a larger clinical study investigating the pharmacokinetics and pharmacodynamics of AL in HIV-infected and HIV-uninfected children and pregnant women in Uganda. HIV-uninfected non-pregnant adults were enrolled as the control group. Major findings from the parent study were published elsewhere, including clinical parameters relating to study drug administration and tolerance [9–11]. Here we report, for the first time, LF concentrations from simultaneously collected venous and capillary plasma samples at 2, 24, and 120hr post last dose in 152 children, 44 pregnant women, 32 non-pregnant adults. Unpaired data were caused by either missing samples or below the limit of quantification (BLQ) (Table A in S1 File). Two samples were excluded as outliers (Table B and C in S1 File). In both cases one of the data pair was BLQ but the other was over 600 ng/mL, which is unlikely to occur. As expected, No BLQ data were found at 2hr and 24hr. For the day 7 (120hr) LF samples, 5 venous samples and 7 capillary samples were BLQ in children, representing 3.5% and 5% of the total samples measured in children for each type of plasma, respectively. In pregnant women, only one capillary sample was BLQ (2.4%). No BLQ sample was found in non-pregnant adults. The reasons for missing samples were 1) failed to access venous blood due to blockage of cannula, 2) missed or delayed sampling time, 3) participants withdrawn from the study.

### Correlation of capillary versus venous plasma concentration of lumefantrine in children

**Fig 1:**
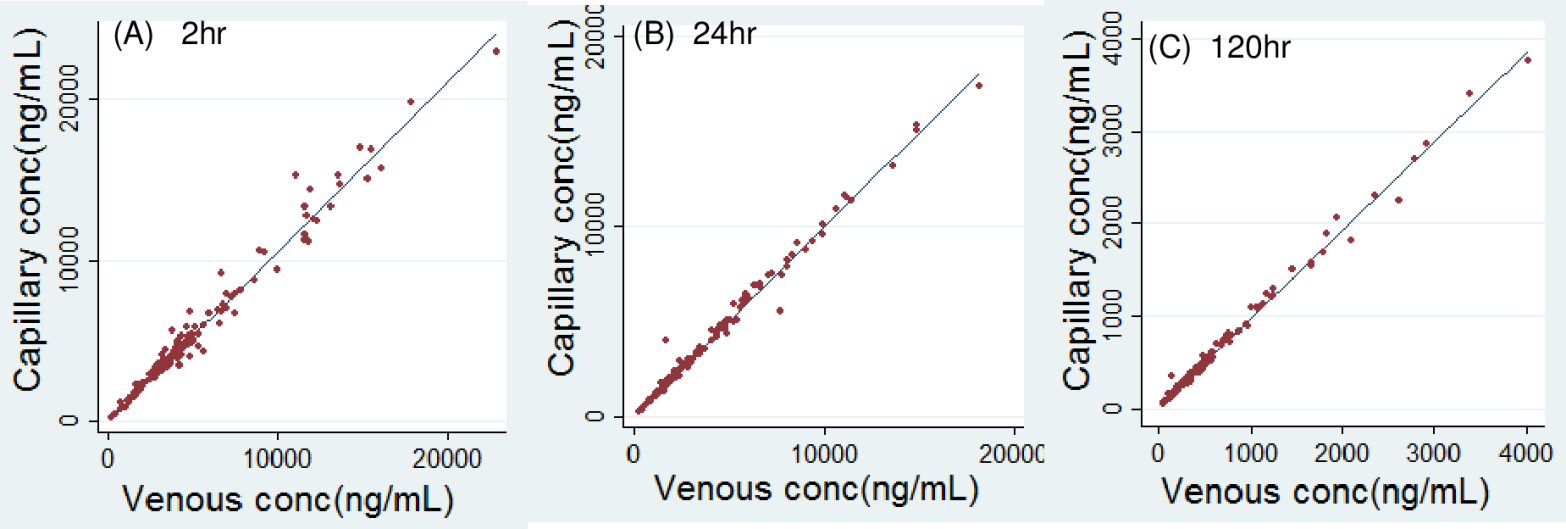
**Linear regression of capillary versus venous plasma lumefantrine in children at 2hr (left panel), 24hr (middle panel), and 120hr (right panel) post last dose**.

Samples were collected from 152 HIV-infected or HIV-uninfected children with malaria, age range 1.1–8.6 years. A total of 142, 147, and 139 pairs of data points for capillary plasma concentration (C_c_) and venous concentration (C_v_) of LF were obtained at 2, 24, and 120hr post last dose of AL, respectively. The corresponding median (range) concentrations are listed in Table 1. With simple linear regression, a good correlation was found at all 3 time intervals (Table 1 and Fig 1). The slopes were 1.05, 0.995, and 0.958 at 2, 24, and 120hr, respectively. The null hypothesis is there is no correlation between capillary and venous LF concentrations (slope = 0). The p-values for slopes of the three equations were all below 0.001, demonstrating a strong correlation between the two groups of LF concentration. Although there is an intercept, the 95% confidence intervals (CI) of the intercepts at 2hr and 24 hr both included zero, and the p-values were 0.29 and 0.18, respectively, suggesting the difference from zero was not statistically significant. The difference between zero and the intercept of equation at 120hr was statistically significant (p = 0.002) but the value is small (18.6), which is below the LLOQ (50ng/mL) and may arise from variation of drug measurement. The results suggest a nearly 1:1 ratio of capillary versus venous LF levels in children. The coefficient of determination (R^2^) from simple linear regression analysis was 0.977, 0.990, and 0.994 at 2, 24, and 120hr, respectively. This is to say, 97.7% of the data at 2hr could be explained by the linear regression equation at 2hr, 99.0% data at 24hr and 99.4% data at 120hr could be explained by the corresponding equations. With natural logarithm transformed concentrations, the R^2^ was 0.982, 0.988, and 0.987, respectively. Overall, better fit of data was found with simple linear regression.

**Table 1:** Correlation of capillary and venous plasma concentration of lumefantrine in children at 2, 24, 120hr post last dose of artemether-lumefantrine.

	2 hr (n = 142)	24 hr (n = 147)	120 hr (n = 139)
Median (range) C_v_, ng/mL	4020 (199, 22900)	2640 (226, 18200)	356 (50.0, 4020)
Median (range) C_c_, ng/mL	4195 (192, 23000)	2640 (250, 17400)	375 (53.7, 3760)
Correlation parameters (C_c_ = a* C_v_ + b)			
slope (95%CI)	1.05 (1.02, 1.08)	0.995 (0.978, 1.01)	0.958 (0.945, 0.972)
intercept (95%CI)	95.0 (-80.4, 270)	56.7(-25.9, 139)	18.6 (6.98, 30.2)
R^2^	0.977	0.990	0.994

Note: C_v_, venous plasma concentration of lumefantrine; C_c_, capillary plasma concentration of lumefantrine; CI, confidence interval; hr, hour; R, correlation coefficient; R^2^, coefficient of determination.

To check the impact of HIV status on correlation of capillary versus venous LF, we performed correlation analysis with subgroups of children with or without HIV infection. There were 59 HIV uninfected children contributing 53, 56, and 53 pairs of data at 2, 24, and 120hr post last dose of AL. With simple linear regression, the slope s (95%CI) were 1.06 (1.01, 1.12), 0.999 (0.943, 1.05), and 0.952 (0.930, 0.974), and the intercepts (95%CI) were 53.5 (-241, 348), 59.8 (-126, 246), and 17.7 (6.12, 29.2), corresponding to 2, 24, and 120hr, respectively, suggesting a linear 1:1 ratio relationship of capillary versus venous LF in HIV uninfected children. There were 93 HIV infected children under either efavirenz, lopinavir/ritonavir, or nevirapine-based antiretroviral therapy. Results from subgroup analysis are consistent with those from combined data (Section 1 in S1 Subgroup Analysis).

### Correlation of capillary versus venous plasma concentration of lumefantrine in pregnant women

**Fig 2:**
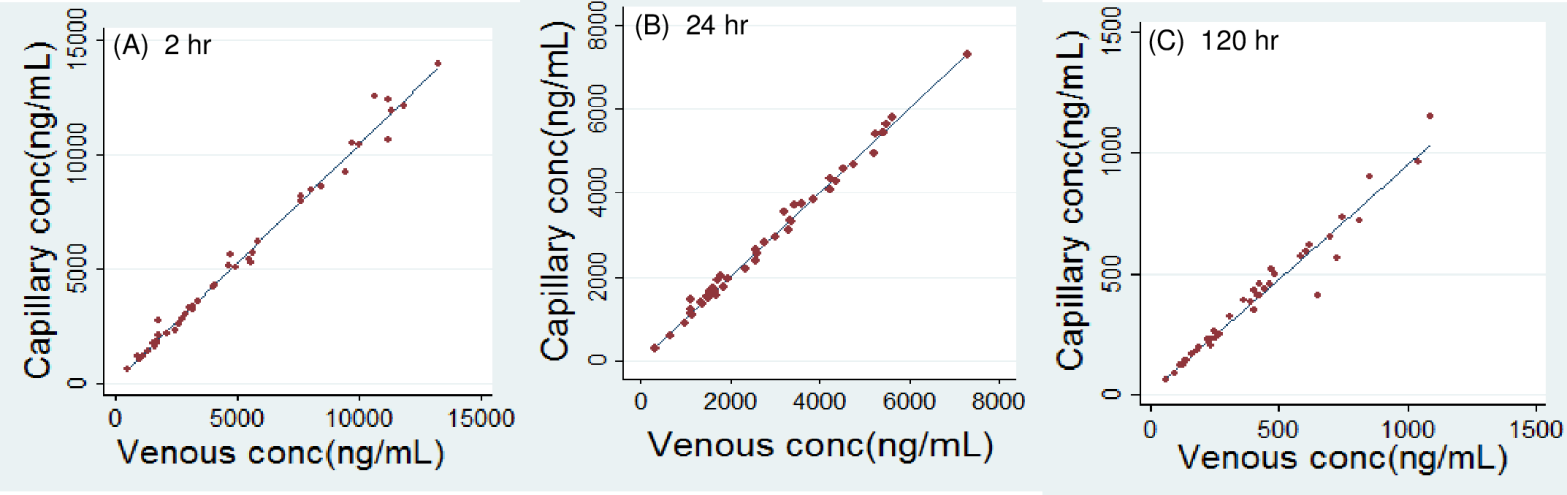
**Linear regression of capillary versus venous plasma lumefantrine in pregnant women at 2hr (left panel), 24hr (middle panel), and 120hr (right panel) post last dose**.

Samples were collected from 44 HIV-infected or HIV-uninfected pregnant women with malaria, ages 18–39 years (Table 2 and Fig 2). A simple linear regression without data transformation gave a good correlation at all 3 time intervals. The slopes were 1.04, 0.997, and 0.941 at 2, 24, and 120hr, respectively, and the p-values for slopes were all below 0.001. The 95%CI of intercepts at 2, 24, and 120hr all included zero, suggesting the intercepts could be zero. The p-values for intercepts at 2, 24, and 120hr were 0.51, 0.40, 0.47, respectively, also suggesting the intercepts were not statistically different from zero. Results suggest a 1:1 ratio of capillary versus venous LF levels in pregnant women. The coefficient of determination (R^2^) was 0.990, 0.993, and 0.958 at 2, 24, and 120hr, respectively, demonstrating over 95% data could be explained by the equations. With natural logarithm transformed concentrations, the R^2^ was 0.989, 0.989, and 0.979, respectively.

**Table 2:** Correlation of capillary and venous plasma concentration of lumefantrine in pregnant women at 2, 24, 120 hr post last dose of artemether-lumefantrine.

	2 hr (n = 43)	24 hr (n = 43)	120 hr (n = 41)
Median (range) C_v_, ng/mL	4040 (537, 13200)	2570 (310, 7310)	389 (60.3, 1090)
Median (range) C_c_, ng/mL	4220 (612, 13900)	2560 (299, 7280)	385 (61.8, 1150)
Correlation parameters (C_c_ = a* C_v_ + b)			
slope (95%CI)	1.04 (1.01, 1.07)	0.997 (0.970, 1.02)	0.941 (0.878, 1.00)
intercept (95%CI)	68.1 (-138, 275)	37.3(-51.0, 126)	11.1 (-19.5, 41.7)
R^2^	0.990	0.993	0.958

Note: C_v_, venous plasma concentration of lumefantrine; C_c_, capillary plasma concentration of lumefantrine; CI, confidence interval; hr, hour; R, correlation coefficient; R^2^, coefficient of determination.

To check the impact of HIV status on correlation of capillary versus venous LF in pregnant women, we also performed subgroup correlation analysis. The subgroup of HIV uninfected pregnant women contains 31 participants contributing 31, 30, and 30 pairs of data at 2, 24, and 120hr post last dose of AL. With simple linear regression, the slope s (95%CI) were 1.04 (0.996, 1.09), 1.01 (0.980, 1.04), and 0.958 (0.881, 1.04), and the intercepts (95%CI) were 73.5 (-240, 387), -1.95 (-110, 106), and 6.27 (-33.6, 46.2), corresponding to 2, 24, and 120hr, respectively, suggesting a linear 1:1 ratio relationship of capillary versus venous LF in HIV uninfected pregnant women. Sample size for HIV infected pregnant women was small (n = 12, 13, 11 at 2, 24, and 120hr, respectively), which reduced the power of analysis, but we still observed a nearly 1:1 ratio of linear correlation except for at 120hr where the slope was 0.835 (Section 2 in S1 Subgroup Analysis).

### Correlation of capillary versus venous plasma concentration of lumefantrine in non-pregnant adults

**Fig 3:**
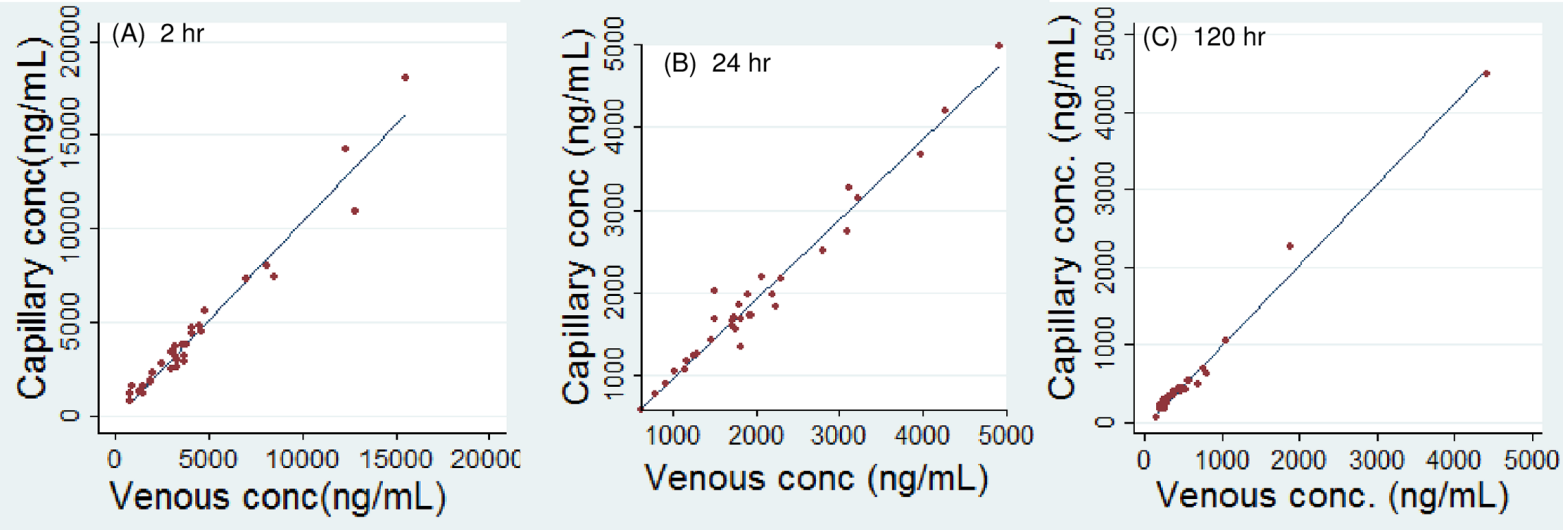
**Linear regression of capillary versus venous plasma lumefantrine in non-pregnant adults at 2hr (left panel), 24hr (middle panel), and 120hr (right panel) post last dose**.

Samples were collected from 32 malaria-infected adults with age range of 16–68 years, including 20 women and 12 men. The results are summarized in Table 3 and Fig 3. Again, a linear regression without data transformation gave a good correlation at all 3 time intervals. The slopes were 1.05, 0.962, and 1.04 at 2, 24, and 120hr, respectively, and the p-values for slopes were all below 0.001, suggesting a strong correlation. The 95% CI of intercepts from all 3 equations included zero, suggesting the intercepts could be zero. The p-values for intercepts at 2, 24, and 120hr were 0.60, 0.90, 0.054, respectively, also suggesting the intercepts were not statistically distinguishable from zero. The coefficient of determination (R^2^) at 2, 24, and 120hr was 0.960, 0.965, and 0.989, respectively, versus 0.945, 0.957, and 0.946, respectively with the log-transformed concentrations, demonstrating an excellent fit of the data with the simple linear regression models, and a 1:1 ratio of capillary versus venous LF levels in non-pregnant adults. Correlation analysis with subgroups of man and women in non-pregnant adults yields similar results (Section 3 in S1 Subgroup Analysis).

**Table 3:** Correlation of capillary and venous plasma concentration of lumefantrine in non-pregnant adults at 2, 24, and 120hr post last dose of artemether-lumefantrine.

	2 hr (n = 32)	24 hr (n = 32)	120 hr (n = 31)
Median (range) C_v_, ng/mL	3315 (742, 15500)	1800 (627, 4930)	382 (143, 4430)
Median (range) C_c_, ng/mL	3290 (769, 18100)	1720 (590, 4990)	356 (70.6, 4480)
Correlation parameters (C_c_ = a* C_v_ + b)			
slope (95%CI)	1.05 (0.972, 1.13)	0.962 (0.893, 1.03)	1.04 (0.996, 1.08)
intercept (95%CI)	-117 (-564, 331)	9.21 (-146, 164)	-40.1 (-80.9, 0.691)
R^2^	0.960	0.965	0.989

Note: C_v_, venous plasma concentration of lumefantrine; C_c_, capillary plasma concentration of lumefantrine; CI, confidence interval; hr, hour; R, correlation coefficient; R^2^, coefficient of determination.

## Discussion

Pharmacokinetic studies are essential for the development of safe and effective antimalarial treatment regimens and battles against drug resistance. Traditionally, venous blood samples are taken for pharmacokinetic assessment, and the vast majority of published pharmacokinetic data has been based on venous plasma samples. However, the large blood volumes and clinical infrastructure needed to perform venous blood sampling for such studies is not practical in the most challenging situations in the tropics, such as in rural areas or in children. New approaches using less invasive capillary sampling coupled with highly sensitive assays on small blood volumes are attractive alternative approaches [14, 15]. In order to facilitate future collections, and to inform comparison of capillary and venous studies from previous published data, we performed a comprehensive correlation study out to day 7 following treatment with AL.

LF is very lipophilic, its octanol-water partition coefficient [logP] is 8.34 [16]. LF is 99.7% bound to proteins in human blood [17], absorbed slowly (about 2hr lag-time and 18hr for complete absorption) and its absorption is enhanced by food intake [18]. The peak concentration is found at 6-8hr post dose [19, 20]. The reported elimination half-life of LF has been 3–7 days [9, 10, 21–23]. Its unique pharmacokinetic properties lead to the concern that capillary concentrations may differ from venous concentrations. Indeed, piperaquine, another long-acting partner drug used in artemisinin combination therapies for malaria, has demonstrated differing levels in samples from the two different blood compartments. One study reported piperaquine levels in capillary plasma were 1.6–1.8 fold higher than those in venous plasma at day 7 post dose in children of 2–10 years-old [24]. A second study reported that piperaquine levels in capillary blood were 1.66-fold higher (90% range 0.92–3.03) than venous blood [5]. LF is also expected to be higher in capillary than venous plasma samples due to its high protein binding and long elimination half-life. However, one published study demonstrated that capillary concentration of LF is similar to venous concentrations [6]. Although a good linear correlation between the 2 types of samples was found with a slope of 0.95 and intercept of 0.52, the regression model required logarithmical transformation of the data, and the study population were limited to patients aged from 15–75 years old with exclusion of pregnant or lactating women. A more recent study reported a 11.9% lower capillary LF concentration than venous concentration based on a population pharmacokinetic modeling approach, as the two types of samples were not simultaneously collected from the same patients [7].

The study reported here was performed with three different populations: children, pregnant women and non-pregnant adults, evaluating impact of physiological distinction on the correlation of capillary versus venous LF. Capillary and venous samples were collected simultaneously from the same patients. Correlation analysis was done at three different time intervals (2, 24, and 120hr post last dose), representing absorption, distribution, and elimination phases, and a simple linear regression model was applied. Compared to regression model with log-transformed data, a simple linear regression gave the same or better coefficient of determination. The relationship between capillary and venous concentrations may be different in different patient demographics, where changes in blood volume, protein/enzymes contents, etc. occur over the course of development and pregnancy. In addition, from recent studies of piperaquine [5], it is plausible that relationship may change over time due to the differing effects of absorption, distribution, and elimination. However, we did not find difference in correlation from these populations and pharmacokinetic phases. It is worth noting that LF accumulated from previous 5 doses added ambiguity to the PK phases and the peak LF ranged from 0 to 8hr in this study. Under the influence of ART treatment, the correlation at day 7 (elimination phase) seems to be off a 1:1 ratio with a 5–15% lower capillary concentration, this difference is not statistically significant and likely caused by smaller sample size and bigger assay variation at lower concentrations. One limitation of this study is that the correlation was evaluated in patient with uncomplicated *P*. *falciparum* malaria. Correlation in healthy subjects and patients infected with other malarial parasites has not been evaluated. In addition, correlation in this study was done through day 7, considering the long half-life of LF, it will be interesting to further study correlation at the tail end of elimination phase. In summary, our results revealed a 1:1 ratio of capillary and venous LF concentrations in all three different populations at 3 different times post dose. Therefore, LF levels measured in capillary plasma samples can be used interchangeably with those measured in venous plasma samples. We conclude that capillary plasma can be an alternative sample collection method for LF in the field without the need of conversion of capillary LF levels to venous LF levels, which facilitates clinical study in resource-limited settings.

The data underlying the presented results can be found in S1 File.

## Supporting information

S1 File Sample profile (Table A) and raw concentration data used for the correlation analysis (Table B and C).
(XLSX)

S1 Subgroup Analysis Subgroup analysis. Section 1, Subgroup analysis based on HIV status in Children; Section 2, Subgroup analysis based on HIV status in pregnant women; Section 3, Subgroup analysis based on sex in non-pregnant adults.
(DOCX)

## References

[1] Olliaro PL, Trigg PI. Status of antimalarial drugs under development. Bull World Health Organ. 1995;73(5):565–71. PubMed Central PMCID: PMCPMC2486827. 8846482 PMC2486827

[2] World Health Organization. Guidelines for the Treatment of Malaria—Third Edition. Geneva, Switzerland: 2015.

[3] Bigira V, Kapisi J, Clark TD, Kinara S, Mwangwa F, Muhindo MK, et al Protective efficacy and safety of three antimalarial regimens for the prevention of malaria in young Ugandan children: a randomized controlled trial. PLoS Med. 2014;11(8):e1001689 10.1371/journal.pmed.1001689 ; PubMed Central PMCID: PMCPMC4122345. 25093754 PMC4122345

[4] Achan J, Kakuru A, Ikilezi G, Ruel T, Clark TD, Nsanzabana C, et al Antiretroviral agents and prevention of malaria in HIV-infected Ugandan children. N Engl J Med. 2012;367(22):2110–8. 10.1056/NEJMoa1200501 ; PubMed Central PMCID: PMCPMC3664297. 23190222 PMC3664297

[5] Ashley EA, Stepniewska K, Lindegardh N, Annerberg A, Tarning J, McGready R, et al Comparison of plasma, venous and capillary blood levels of piperaquine in patients with uncomplicated falciparum malaria. Eur J Clin Pharmacol. 2010;66(7):705–12. 10.1007/s00228-010-0804-7 ; PubMed Central PMCID: PMCPMC2883082. 20300743 PMC2883082

[6] van Vugt M, Ezzet F, Phaipun L, Nosten F, White NJ. The relationship between capillary and venous concentrations of the antimalarial drug lumefantrine (benflumetol). Trans R Soc Trop Med Hyg. 1998;92(5):564–5. . 9861382 10.1016/s0035-9203(98)90917-8

[7] Kloprogge F, Piola P, Dhorda M, Muwanga S, Turyakira E, Apinan S, et al Population Pharmacokinetics of Lumefantrine in Pregnant and Nonpregnant Women With Uncomplicated Plasmodium falciparum Malaria in Uganda. CPT Pharmacometrics Syst Pharmacol. 2013;2:e83 10.1038/psp.2013.59 ; PubMed Central PMCID: PMCPMC3852159. 24226803 PMC3852159

[8] Antimalarial Pharmacology in Children and Pregnant Women in Uganda [Internet]. [cited July 8, 2016]. Available from: https://clinicaltrials.gov/ct2/show/NCT01717885?term=aweeka&rank=7.

[9] Parikh S, Kajubi R, Huang L, Ssebuliba J, Kiconco S, Gao Q, et al Antiretroviral Choice for HIV Impacts Antimalarial Exposure and Treatment Outcomes in Ugandan Children. Clinical infectious diseases: an official publication of the Infectious Diseases Society of America. 2016;63(3):414–22. Epub 2016/05/05. 10.1093/cid/ciw291 ; PubMed Central PMCID: PMCPmc4946019. 27143666 PMC4946019

[10] Nyunt MM, Nguyen VK, Kajubi R, Huang L, Ssebuliba J, Kiconco S, et al Artemether-Lumefantrine Pharmacokinetics and Clinical Response Are Minimally Altered in Pregnant Ugandan Women Treated for Uncomplicated Falciparum Malaria. Antimicrobial agents and chemotherapy. 2015;60(3):1274–82. 10.1128/AAC.01605-15 ; PubMed Central PMCID: PMCPMC4775973. 26666942 PMC4775973

[11] Kajubi R, Huang L, Were M, Kiconco S, Li F, Marzan F, et al Parasite Clearance and Artemether Pharmacokinetics Parameters Over the Course of Artemether-Lumefantrine Treatment for Malaria in Human Immunodeficiency Virus (HIV)-Infected and HIV-Uninfected Ugandan Children. Open Forum Infect Dis. 2016;3(4):ofw217 10.1093/ofid/ofw217 ; PubMed Central PMCID: PMCPMC5170492. 28018925 PMC5170492

[12] Huang L, Li X, Marzan F, Lizak PS, Aweeka FT. Determination of lumefantrine in small-volume human plasma by LC-MS/MS: using a deuterated lumefantrine to overcome matrix effect and ionization saturation. Bioanalysis. 2012;4(2):157–66. 10.4155/bio.11.303 ; PubMed Central PMCID: PMCPMC3321395. 22250798 PMC3321395

[13] Huang L, Lizak PS, Jayewardene AL, Marzan F, Lee MN, Aweeka FT. A modified method for determination of lumefantrine in human plasma by HPLC-UV and combination of protein precipitation and solid-phase extraction: application to a pharmacokinetic study. Anal Chem Insights. 2010;5:15–23. ; PubMed Central PMCID: PMCPMC2865164. 20448843 10.4137/aci.s4431 PMC2865164

[14] Simpson JA, Aarons L, White NJ. How can we do pharmacokinetic studies in the tropics? Trans R Soc Trop Med Hyg. 2001;95(4):347–51. Epub 2001/10/03. . 11579871 10.1016/s0035-9203(01)90178-6

[15] Davis TM. Pharmacokinetic studies of antimalarials: recent developments. Expert review of clinical pharmacology. 2016;9(3):341–3. Epub 2015/10/30. 10.1586/17512433.2016.1108190 . 26512938

[16] Wahajuddin, Raju KS, Singh SP, Taneja I. Investigation of the functional role of P-glycoprotein in limiting the oral bioavailability of lumefantrine. Antimicrobial agents and chemotherapy. 2014;58(1):489–94. 10.1128/AAC.01382-13 ; PubMed Central PMCID: PMC3910766. 24189249 PMC3910766

[17] Colussi D, Parisot C, Legay F, Lefevre G. Binding of artemether and lumefantrine to plasma proteins and erythrocytes. European journal of pharmaceutical sciences: official journal of the European Federation for Pharmaceutical Sciences. 1999;9(1):9–16. . 10493991 10.1016/s0928-0987(99)00037-8

[18] White NJ, van Vugt M, Ezzet F. Clinical pharmacokinetics and pharmacodynamics of artemether-lumefantrine. Clin Pharmacokinet. 1999;37(2):105–25. . 10496300 10.2165/00003088-199937020-00002

[19] Byakika-Kibwika P, Lamorde M, Okaba-Kayom V, Mayanja-Kizza H, Katabira E, Hanpithakpong W, et al Lopinavir/ritonavir significantly influences pharmacokinetic exposure of artemether/lumefantrine in HIV-infected Ugandan adults. J Antimicrob Chemother. 2012;67(5):1217–23. 10.1093/jac/dkr596 ; PubMed Central PMCID: PMCPMC3324422. 22316571 PMC3324422

[20] Novartis Pharm. Co. Coartem: full prescribing information [updated January, 2018; cited 2018 May 15]. Available from: https://www.pharma.us.novartis.com/sites/www.pharma.us.novartis.com/files/coartem.pdf.

[21] German P, Parikh S, Lawrence J, Dorsey G, Rosenthal PJ, Havlir D, et al Lopinavir/ritonavir affects pharmacokinetic exposure of artemether/lumefantrine in HIV-uninfected healthy volunteers. J Acquir Immune Defic Syndr. 2009;51(4):424–9. 10.1097/QAI.0b013e3181acb4ff . 19506482

[22] Huang L, Parikh S, Rosenthal PJ, Lizak P, Marzan F, Dorsey G, et al Concomitant efavirenz reduces pharmacokinetic exposure to the antimalarial drug artemether-lumefantrine in healthy volunteers. J Acquir Immune Defic Syndr. 2012;61(3):310–6. 10.1097/QAI.0b013e31826ebb5c ; PubMed Central PMCID: PMCPMC3511816. 22918158 PMC3511816

[23] Djimde A, Lefevre G. Understanding the pharmacokinetics of Coartem. Malar J. 2009;8 Suppl 1:S4 10.1186/1475-2875-8-S1-S4 ; PubMed Central PMCID: PMCPMC2760239. 19818171 PMC2760239

[24] Zongo I, Some FA, Somda SA, Parikh S, Rouamba N, Rosenthal PJ, et al Efficacy and day 7 plasma piperaquine concentrations in African children treated for uncomplicated malaria with dihydroartemisinin-piperaquine. PLoS One. 2014;9(8):e103200 10.1371/journal.pone.0103200 ; PubMed Central PMCID: PMCPMC4136730. 25133389 PMC4136730

