# Supplementary material for: Strong correlation of lumefantrine concentrations in capillary and venous plasma from malaria patients
Source: PLoS One. 2018 Aug 16;13(8):e0202082. doi: 10.1371/journal.pone.0202082 (PMC6095545; doi:10.1371/journal.pone.0202082)
Supplement: S1 Subgroup Analysis — Section 1, Subgroup analysis based on HIV status in Children; Section 2, Subgroup analysis based on HIV status in pregnant women; Section 3, Subgroup analysis based on sex in non-pregnant adults. (DOCX) [file pone.0202082.s002.docx]

Subgroup analysis

- 1. Subgroup analysis based on HIV status in children
  2. HIV negative children

|  | 2 hr (n=53)* | 24 hr (n=56)** | 120 hr (n=53)*** |
| --- | --- | --- | --- |
| Median (range) C_v_, ng/mL | 4260 (199, 13600) | 2280 (293, 9030) | 333 (161, 1800) |
| Median (range) C_c_, ng/mL | 4270 (192, 15300) | 2365 (294, 8790) | 330 (154, 1690) |
| Correlation parameters (C_c_ =a* C_v_ + b) | | | |
| slope (95%CI) | 1.06 (1.01, 1.12)  P<0.001 | 0.999 (0.943, 1.05)  P<0.001 | 0.952 (0.930, 0.974)  P<0.001 |
| intercept (95%CI) | 53.5 (-241, 348)  P=0.72 | 59.8(-126, 246)  P=0.52 | 17.7 (6.12, 29.2)  P=0.003 |
| R^2^ | 0.967 | 0.960 | 0.993 |

*n=58 for capillary LF; ** n=57 for Venous LF and 58 for capillary LF; ***n=54 for capillary LF. Cv, venous plasma concentration of lumefantrine; Cc, capillary plasma concentration of lumefantrine; CI, confidence interval; R, correlation coefficient.

1.2. HIV positive children

This subgroup of children were all HIV positive but treated with one of the 3 antiretrovial therapies: lopinavir-ritonavir-, nevirapiine-, and efavirenz-based antiretroviral therapies. This analysis combined children treated with all 3 different regimens.

|  | 2 hr (n=89)* | 24 hr (n=91)** | 120 hr (n=86)*** |
| --- | --- | --- | --- |
| Median (range) C_v_, ng/mL | 3690 (343, 22900) | 2870 (226, 18200) | 359 (50.0, 4020) |
| Median (range) C_c_, ng/mL | 3780 (360, 23000) | 3060 (250, 17400) | 378 (53.7, 3760) |
| Correlation parameters (C_c_ =a* C_v_ + b) | | | |
| slope (95%CI) | 1.05 (1.02, 1.08)  P<0.001 | 0.995 (0.978, 1.01)  P<0.001 | 0.958 (0.941, 0.975)  P<0.001 |
| intercept (95%CI) | 97.2 (-137, 331)  P=0.41 | 46.1(-54.6, 147)  P=0.37 | 21.1 (3.25, 39.0)  P=0.021 |
| R^2^ | 0.979 | 0.993 | 0.993 |

*n=91 for venous LF; ** n=93 for Venous LF; ***n=88 for venous LF and 87 for capillary LF.

1.2.1 HIV positive children on lopinavir-ritonavir based antiretroviral treatment

There are 31 children in this subgroup.

|  | 2 hr (n=30) | 24 hr (n=31) | 120 hr (n=31) |
| --- | --- | --- | --- |
| Median (range) C_v_, ng/mL | 5160 (1680, 22900) | 6600 (824, 18200) | 1140 (65.0, 4020) |
| Median (range) C_c_, ng/mL | 5235 (1700, 23000) | 6820 (788, 17400) | 1130 (69.8, 3760) |
| Correlation parameters (C_c_ =a* C_v_ + b) | | | |
| slope (95%CI) | 1.05 (1.00, 1.10)  P<0.001 | 0.985 (0.942, 1.03)  P<0.001 | 0.937 (0.902, 0.972)  P<0.001 |
| intercept (95%CI) | 38.2 (-427, 503)  P=0.87 | 117(-224, 457)  P=0.49 | 66.9 (8.64, 125)  P=0.03 |
| R^2^ | 0.986 | 0.987 | 0.991 |

1.2.2. HIV positive children on nevirapine-based antiretroviral treatment

|  | 2 hr (n=29)* | 24 hr (n=30) | 120 hr (n=30) |
| --- | --- | --- | --- |
| Median (range) C_v_, ng/mL | 3315 (658, 13100) | 3495 (655, 14900) | 401 (120, 1240) |
| Median (range) C_c_, ng/mL | 3540 (700, 13300) | 3565 (670, 15100) | 414 (111, 1210) |
| Correlation parameters (C_c_ =a* C_v_ + b) | | | |
| slope (95%CI) | 0.986 (0.918, 1.05)  P<0.001 | 1.00 (0.974, 1.03)  P<0.001 | 1.00 (0.955, 1.05)  P<0.001 |
| intercept (95%CI) | 303 (-50.4, 657)  P=0.09 | 22.0(-117, 161)  P=0.75 | -2.42 (-26.0, 21.2)  P=0.84 |
| R^2^ | 0.971 | 0.995 | 0.985 |

*n=30 for venous LF.

1.2.3. HIV positive children on efavirenz-based antiretroviral treatment

|  | 2 hr (n=30)* | 24 hr (n=30)** | 120 hr (n=25)*** |
| --- | --- | --- | --- |
| Median (range) C_v_, ng/mL | 2850 (343, 15300) | 1255 (226, 6110) | 116 (50, 515) |
| Median (range) C_c_, ng/mL | 2880 (360, 15300) | 1425 (250, 6310) | 114 (53.7, 479) |
| Correlation parameters (C_c_ =a* C_v_ + b) | | | |
| slope (95%CI) | 1.09 (1.01, 1.17)  P<0.001 | 1.04 (1.02, 1.05)  P<0.001 | 0.902 (0.841, 0.963)  P<0.001 |
| intercept (95%CI) | 21.5 (-446, 489)  P=0.93 | -38.0(-80.4, 4.29)  P=0.08 | 13.0 (0.25, 25.8)  P=0.05 |
| R^2^ | 0.963 | 0.998 | 0.976 |

*n=31 for venous LF; **n=32 for venous LF; ***n=27 for venous LF and 26 for capillary LF.

1. Subgroup analysis based on HIV status in pregnant women
   1. HIV negative pregnant women.

|  | 2 hr (n=31) | 24 hr (n=30) | 120 hr (n=30) |
| --- | --- | --- | --- |
| Median (range) C_v_, ng/mL | 4950 (991, 13200) | 3100 (662, 7310) | 409 (60.3, 1090) |
| Median (range) C_c_, ng/mL | 5280 (1060, 13900) | 3030 (614, 7280) | 406 (61.8, 1150) |
| Correlation parameters (C_c_ =a* C_v_ + b) | | | |
| slope (95%CI) | 1.04 (0.996, 1.09), p<0.001 | 1.01 (0.980, 1.04), p<0.001 | 0.958 (0.881, 1.04)  P<0.001 |
| intercept (95%CI) | 73.5(-240, 387), p=0.64 | -1.95(-110, 106), p=0.97 | 6.27 (-33.6, 46.2)  p=0.75 |
| R^2^ | 0.987 | 0.994 | 0.959 |

- 1. HIV positive pregnant women.

|  | 2 hr (n=12)* | 24 hr (n=13) | 120 hr (n=11) |
| --- | --- | --- | --- |
| Median (range) C_v_, ng/mL | 2130 (537, 7610) | 1710 (310, 5200) | 253 (92.3, 726) |
| Median (range) C_c_, ng/mL | 2480 (612, 8140) | 1750 (299, 4930) | 236 (88.4, 588) |
| Correlation parameters (C_c_ =a* C_v_ + b) | | | |
| slope (95%CI) | 1.04 (0.993, 1.08), p<0.001 | 0.957 (0.890, 1.02), p<0.001 | 0.835 (0.700, 0.971)  p<0.001 |
| intercept (95%CI) | 70.2(-83.6, 224), p=0.33 | 117(-59.2, 293), p=0.17 | 35.9 (-13.5, 85.3)  p=0.14 |
| R^2^ | 0.996 | 0.989 | 0.956 |

N=13 for venous LF.

3. Subgroup analysis based on sex in non-pregnant adults.

3.1. Male participants in the non-pregnant adult group

|  | 2 hr (n=12) | 24 hr (n=12) | 120 hr (n=11) |
| --- | --- | --- | --- |
| Median (range) C_v_, ng/mL | 3075 (750, 8140) | 1720 (627, 2810) | 382 (189, 4430) |
| Median (range) C_c_, ng/mL | 2825 (769, 8040) | 1635 (590, 2500) | 356 (181, 4480) |
| Correlation parameters (C_c_ =a* C_v_ + b) | | | |
| slope (95%CI) | 0.950 (0.772, 1.13)  P<0.001 | 0.836 (0.687, 0.985)  p<0.001 | 1.01 (0.978, 1.05)  P<0.001 |
| intercept (95%CI) | 65.0 (-570, 700)  p=0.82 | 147 (-116, 410)  p=0.24 | -31.2 (-81.9, 19.5)  p=0.20 |
| R^2^ | 0.934 | 0.940 | 0.998 |

3.2. Female participants in the non-pregnant adult group.

|  | 2 hr (n=20) | 24 hr (n=20) | 120 hr (n=20) |
| --- | --- | --- | --- |
| Median (range) C_v_, ng/mL | 3905 (742, 15500) | 1910 (793, 4930) | 372 (143, 1890) |
| Median (range) C_c_, ng/mL | 4080 (1130, 18100) | 1910 (784, 4990) | 371 (70.6, 2270) |
| Correlation parameters (C_c_ =a* C_v_ + b) | | | |
| slope (95%CI) | 1.05 (0.947, 1.16)  P<0.001 | 0.958 (0.873, 1.04)  P<0.001 | 1.17 (1.06, 1.27)  P<0.001 |
| intercept (95%CI) | -45.2 (-734, 644)  P=0.89 | 61.0 (-152, 274)  P=0.56 | -99.2 (-165, -33.6)  P=0.005 |
| R^2^ | 0.960 | 0.969 | 0.969 |
